# P-487. [^18^F]-labeled J3 Nanobody is a Specific PET Tracer for Molecular Imaging of HIV-1 gp120

**DOI:** 10.1093/ofid/ofae631.686

**Published:** 2025-01-29

**Authors:** Neysha Martinez-Orengo, Swati Shah, Jianhao Lai, Falguni Basuli, Mitchell Turner, Morteza Peiravi, Kevon Sampson, Rolf Swenson, Frank Maldarelli, Avindra Nath, Chuen-Yen Lau, Dima A Hammoud

**Affiliations:** National Institutes of Health, Bethesda, Maryland; National Institutes of Health, Bethesda, Maryland; National Institutes of Health, Bethesda, Maryland; National Institutes of Health, Bethesda, Maryland; National Institutes of Health, Bethesda, Maryland; National Institutes of Health, Bethesda, Maryland; National Institutes of Health, Bethesda, Maryland; National Institutes of Health, Bethesda, Maryland; National Institutes of Health, Bethesda, Maryland; National Institute of Neurological Disorders and Stroke, Bethesda, MD; National Institutes of Health, Bethesda, Maryland; NIH Clinical Center, Bethesda, MD

## Abstract

**Background:**

Targeted molecular imaging of HIV-1 can potentially identify viral reservoirs and provide a better understanding of disease pathogenesis and management. Nanobody-based radiolabeled probes offer several advantages over full antibodies, including smaller size, deep tissue penetration, and high stability. In this study, three fluorine-18 labeled nanobodies targeting the well-conserved CD4-binding site on HIV-1 envelope protein (gp120) were evaluated in gp120-expressing flank tumor models.

**Figure:**
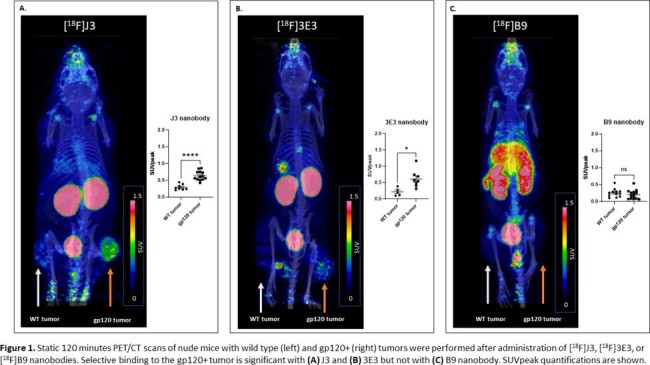

**Methods:**

Binding affinities to gp120 protein were determined by bio-layer interferometry. For *in vivo* PET imaging, an animal model with wild type (WT) and/or gp120-expressing HEK293T cells (gp120+) flank tumors was developed in nude mice. Radiolabeled nanobodies were prepared by an indirect labeling method using 6-fluoronicotinic acid-2,3,5,6-tetrafluorophenyl ester (6-[18F]FPy-TFP) prosthetic group. Dynamic PET/CT images up to 60 minutes and static images at 120 minutes were obtained. Tumors were evaluated e*x vivo* using immunofluorescent staining. Unpaired t test was used for statistical analysis.

**Figure:**
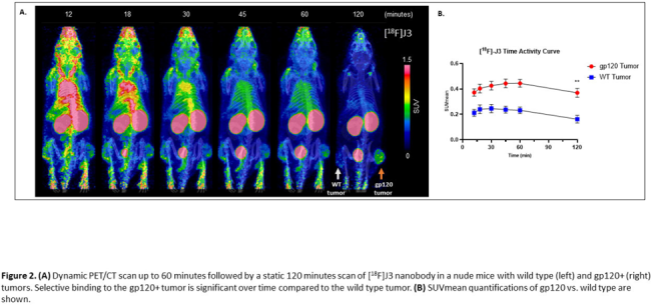

**Results:**

J3 nanobody showed higher binding affinity to gp120 compared to 3E3 and B9, with KD values of 1.1nM, 3.4nM, and 23.7nM, respectively. PET/CT imaging revealed selective binding (measured as SUVpeak) to gp120+ compared to WT tumors with J3 (0.64 ± 0.14 vs. 0.29 ± 0.08) and 3E3 (0.61 ± 0.27 vs. 0.22 ± 0.12) but not with B9 (0.21 ± 0.14 vs 0.25 ± 0.12). After 2 hours, J3 and 3E3 had minimal background signal, whereas B9 showed poor ligand clearance and off target binding (Fig. 1). [^18^F]J3 time activity curves (TACs) from dynamic and static scans showed selective binding to gp120+ tumors compared to WT tumors (Fig. 2). To validate our PET findings, *ex vivo* gp120 tumor expression was confirmed by immunofluorescence staining. J3 nanobody showed specific immunoreactivity that colocalized with VRC01, an anti-gp120 full antibody (Fig. 3).

**Figure:**
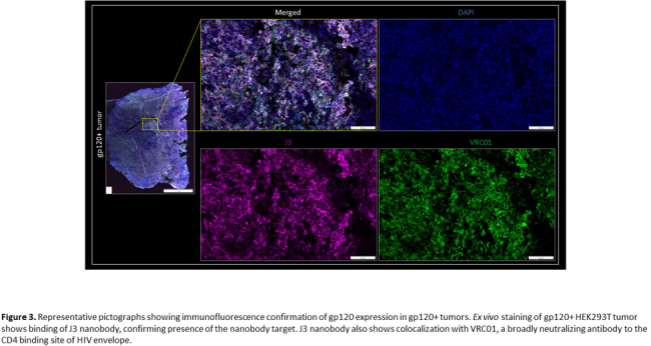

**Conclusion:**

We evaluated the binding characteristics of three 18F-labeled nanobodies to gp120+ cells. Overall, J3 nanobody had higher specificity and binding to its target compared to 3E3 and B9, demonstrating a higher potential for non-invasive identification of HIV-infected cells in infected models.

**Disclosures:**

**All Authors**: No reported disclosures

